# Decellularized Whole-Organ Pre-vascularization: A Novel Approach for Organogenesis

**DOI:** 10.3389/fbioe.2021.756755

**Published:** 2021-10-19

**Authors:** Ibrahim Fathi, Takehiro Imura, Akiko Inagaki, Yasuhiro Nakamura, Ayman Nabawi, Masafumi Goto

**Affiliations:** ^1^ Division of Transplantation and Regenerative Medicine, Tohoku University, Sendai, Japan; ^2^ Department of Surgery, University of Alexandria, Alexandria, Egypt; ^3^ Division of Pathology, Faculty of Medicine, Tohoku Medical and Pharmaceutical University, Sendai, Japan; ^4^ Department of Surgery, Tohoku University, Sendai, Japan

**Keywords:** whole-liver decellularization, arteriovenous, pancreatic islet, extracellular matrix, axial vascularization

## Abstract

**Introduction:** Whole-organ decellularization is an attractive approach for three-dimensional (3D) organ engineering. However, progress with this approach is hindered by intra-vascular blood coagulation that occurs after *in vivo* implantation of the re-cellularized scaffold, resulting in a short-term graft survival. In this study, we explored an alternative approach for 3D organ engineering through an axial pre-vascularization approach and examined its suitability for pancreatic islet transplantation.

**Methods:** Whole livers from male Lewis rats were decellularized through sequential arterial perfusion of detergents. The decellularized liver scaffold was implanted into Lewis rats, and an arteriovenous bundle was passed through the scaffold. At the time of implantation, fresh bone marrow preparation (BM; *n* = 3), adipose-derived stem cells (ADSCs; *n* = 4), or HBSS (*n* = 4) was injected into the scaffold through the portal vein. After 5 weeks, around 2,600 islet equivalents (IEQs) were injected through the portal vein of the scaffold. The recipient rats were rendered diabetic by the injection of 65 mg/kg STZ intravenously 1 week before islet transplantation and were followed up after transplantation by measuring the blood glucose and body weight for 30 days. Intravenous glucose tolerance test was performed in the cured animals, and samples were collected for immunohistochemical (IHC) analyses. Micro-computed tomography (CT) images were obtained from one rat in each group for representation.

**Results:** Two rats in the BM group and one in the ADSC group showed normalization of blood glucose levels, while one rat from each group showed partial correction of blood glucose levels. In contrast, no rats were cured in the HBSS group. Micro-CT showed evidence of sprouting from the arteriovenous bundle inside the scaffold. IHC analyses showed insulin-positive cells in all three groups. The number of von-Willebrand factor-positive cells in the islet region was higher in the BM and ADSC groups than in the HBSS group. The number of 5-bromo-2′-deoxyuridine-positive cells was significantly lower in the BM group than in the other two groups.

**Conclusions:** Despite the limited numbers, the study showed the promising potential of the pre-vascularized whole-organ scaffold as a novel approach for islet transplantation. Both BM- and ADSCs-seeded scaffolds were superior to the acellular scaffold.

## Introduction

Pancreatic islet transplantation is a promising therapy for type 1 diabetes. However, the current standard site for islet transplantation, intraportal transplantation, has several shortcomings. For instance, a large proportion of the injected islets are lost during or shortly after the implantation (Bennet et al., 2000) as a result of instant blood-mediated inflammatory responses, invasion by natural killer cells and low oxygen tension (Smink et al., 2013). Furthermore, the ability of the transplanted cells to maintain tight glycemic control is not sustained in the long term (Hering, 2005). The transplanted islets are also not accessible for follow-up or subsequent removal, which is a concern if regenerative approaches are applied. Therefore, a more suitable alternative site for islet transplantation is desirable and has been the focus of many investigations (Merani et al., 2008; Smink et al., 2013).

Whole-organ decellularization is an approach in which the cellular content of an organ is removed, commonly through vascular perfusion of detergents, leaving an intact extracellular matrix (ECM) that can be recellularized using the native vascular system of the scaffold. This seeded scaffold can then be transplanted using vascular anastomosis techniques. Hypothetically, the merits of this approach include the removal of the antigenic cellular elements from the organ in order to prevent host adverse immune response to the scaffold; using the natural three-dimensional (3D) architecture of the organ formed by the ECM proteins with the incorporated growth factors as a scaffold for cell seeding, seeding cells with a better immunological profile towards the recipient; and using the natural vascular network to deliver oxygen and nutrients to the complex 3D scaffold after anastomosing it to the recipient vasculature (Fathi et al., 2017).

Due to the complex architecture of the liver and its rich vascular supply, such an approach has proven attractive for liver bioengineering. However, despite the evident progress and refinement of this approach over the last 2 decades, a major hurdle remains. The de-endothelialization of the vascular network during decellularization allows for the direct contact of the recipient’s blood and the ECM of the blood vessels after vascular anastomosis in the host. ECM proteins include collagen and laminin, which can initiate the blood coagulation cascade (Bao et al., 2015; Bruinsma et al., 2015), thereby resulting in vascular occlusion due to thrombosis. Indeed, the first trials of *in vivo* transplantation of decellularized or recellularized liver scaffolds were associated with a short-term graft survival (Uygun et al., 2010; Bao et al., 2011; Bruinsma et al., 2015; Ko et al., 2015).

Several research groups have sought to prevent intravascular coagulation through different approaches, including re-endothelialization of the graft to prevent direct contact of blood with ECM (Ko et al., 2015; Kojima et al., 2018); heparin immobilization on the ECM of the scaffold through covalent binding, end-point immobilization, or layer-by-layer techniques (Bao et al., 2015; Bruinsma et al., 2015); and coating the vascular network with heparin-gelatin (Hussein et al., 2016). However, while these approaches did result in the significant prolongation of the graft survival and improved vascular patency to different extents, the long-term survival of a functioning graft has not yet been reported.

Several groups have conversely sought to bypass this hurdle by directly implanting either pieces of the seeded scaffold under the kidney capsule (Wang et al., 2014) or in the subcutaneous tissue (Citro et al., 2019) or the intact seeded scaffold into the peritoneal cavity (Vishwakarma et al., 2019) and relying on peripheral vascularization to supply this relatively small scaffold, sacrificing the native vascular network. In such cases, the ECM scaffold was shown capable of supporting the cell function. However, the problem with this maneuver is the difficulty in scaling it to a clinically meaningful size.

**FIGURE 1:**
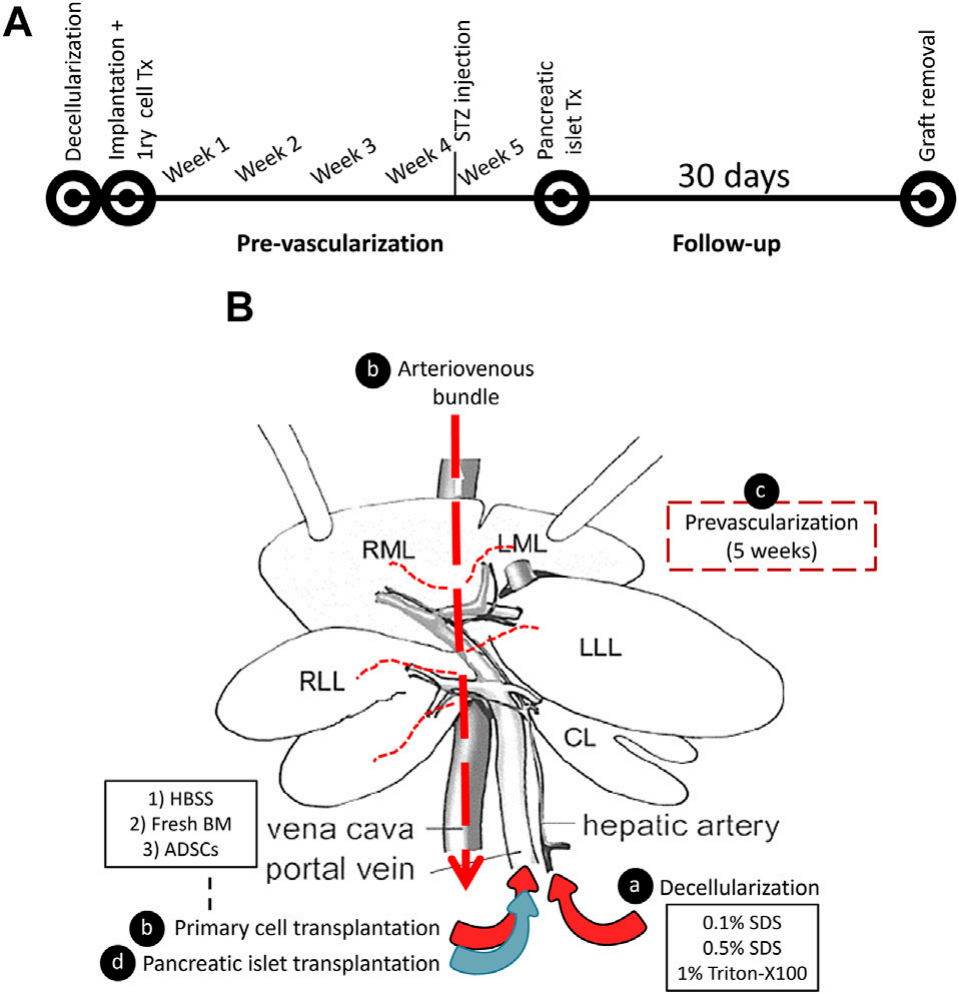
A schematic representation of the decellularized liver pre-vascularization study. **(A)** A timeline representing the flow of the study. **(B)** The graft modulation during the study: **(A)** the rat liver was decellularized by detergent perfusion through the HA, **(B)** the decellularized scaffold was implanted into the recipient, and an AV bundle was threaded through the inferior vena cava using a tube that was placed in the IVC. Then, either fresh BM or ADSC or HBSS vehicle was infused through the PV of the scaffold, **(C)** a five-week period was allowed for pre-vascularization, and **(D)** syngeneic pancreatic islets were transplanted through the PV.

In the present study, we sought an alternative approach to circumvent the current obstacles through scaffold pre-vascularization. Our approach relies on axial vascularization, where a ligation-type arteriovenous (AV) bundle is used for the pre-vascularization of the scaffold while maintaining the natural vascular cues of the graft for cell transplantation and graft modulation (Figure 1).

We herein report our preliminary results of this technique and compare the pre-vascularization of acellular scaffold and seeded scaffold containing either fresh bone marrow preparation (BM) or cultured adipose-derived stem cells (ADSCs), as demonstrated through the function of transplanted pancreatic islets.

## Materials and Methods

### Animals

Lewis rats were purchased from Japan SLC Inc (Shizuoka, Japan). For all experiments, scaffold recipients were 14–16 weeks old, scaffold and pancreatic islet donors were 10–12 weeks old, and BM and ADSC donors were 8 weeks old. Except for the BM donors, all of the rats used in this study were males. All animals had free access to a standard diet and water.

The experiments were approved by the local ethics committee (approved protocol 2019MdA-104) and performed in accordance with national and institutional regulations. Animals were maintained in a specific-pathogen-free environment. All surgeries were performed under anesthesia, and all efforts were made to minimize suffering.

### Rat Whole-Liver Decellularization

Livers were obtained from male Lewis rats after cannulation of the portal vein (PV) and hepatic artery (HA) with a 22-G venflon cannula, and a tube (TOP X2-100 extension tube, TOP Co., Tokyo, Japan) was placed in the infra-hepatic inferior vena cava (IVC-tube).

**FIGURE 2:**
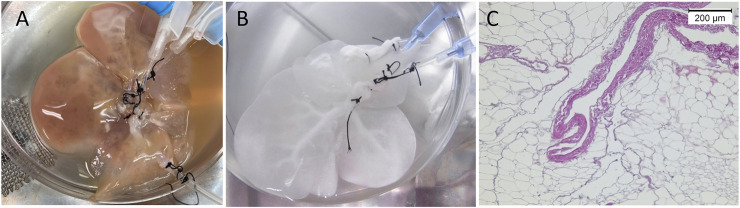
Rat liver decellularization. **(A)** The liver during perfusion of detergents, **(B)** transparent DLM after completion of the decellularization protocol, **(C)** DLM histology showing preservation of the native honeycomb appearance of the liver ECM.

The liver was decellularized following the protocol described by Vishwakarma et al. (2019). In brief, after washing out the blood, the liver was sequentially perfused with 0.1% w/v sodium dedocylsulphate (SDS; Wako, Osaka, Japan) for 2 h, SDS 0.5% w/v for 18 h, distilled water for 10 min, 1% Triton X-100 v/v (Wako) for 30 min, and finally distilled water (DW) for 30 min to wash out the detergents. Perfusion was performed through the HA cannula at a flow rate of 1 ml/min using a peristaltic pump (Perista AC-2110; ATTO Corporation, Tokyo, Japan). The decellularized liver (DLM) was kept in 100 ml of DW containing 1% (1,000 U) heparin sodium (Mochida, Tokyo, Japan) and 1% penicillin/streptomycin (Thermo Fisher Scientific, Waltham, MA, United States) in a sterile container at 4°C until the time of implantation (Figure 2).

All procedures were performed using sterile techniques and sterile DW. Detergent perfusion was performed in a laminar flow cabinet. After completion of decellularization protocol, 0.5 × 0.5-cm samples were excised and fixed in 4% paraformaldehyde for the histological assessment of the decellularization in selected samples.

### ADSC Isolation and Culture

**FIGURE 3:**
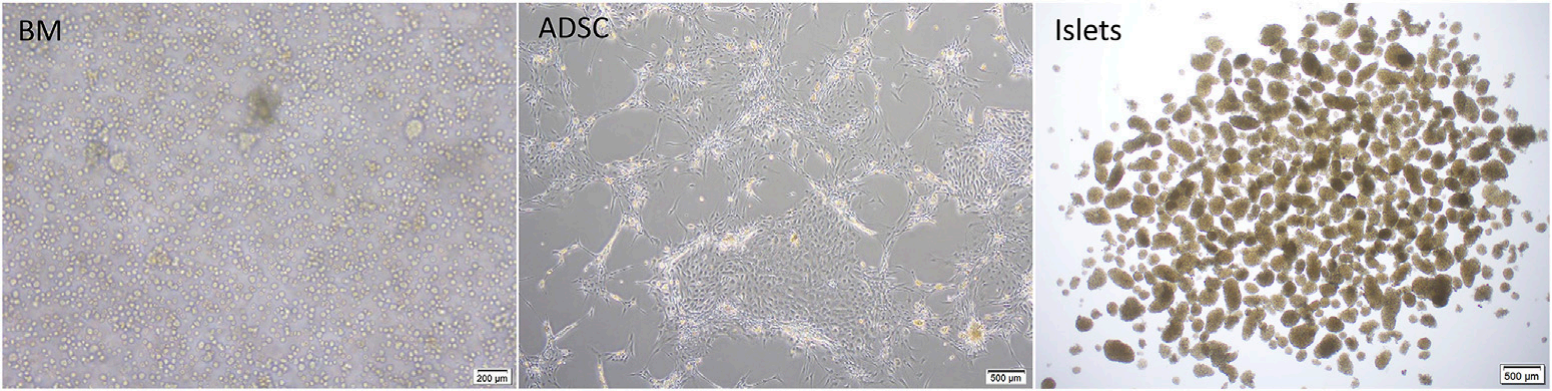
Cell preparations used in the study; BM: fresh rat bone marrow, ADSCs: rat adipose-derived stem cells.

ADSCs were isolated from male Lewis rats as described previously (Ohmura et al., 2010), with a few modifications. In brief, adipose tissue was obtained from the inguinal fat pad and cut into fine pieces before being placed in ice-cold phosphate-buffered saline (PBS; Sigma-Aldrich, St. Louis, MO, United States) and then in Hank’s balanced salt solution (HBSS; Sigma-Aldrich) containing 1 mg/ml collagenase II (Sigma-Aldrich) and 1% penicillin/streptomycin, at 37°C for 30 min with gentle agitation. The digested tissue was filtered through a sterile 70-μm cell-strainer, centrifuged at 400 *g* for 5 min at room temperature, and resuspended. This process was repeated twice. ADSCs were seeded into T-75 cell culture flasks (Thermo Fisher Scientific) in ADSC-1 medium (Kohjin Bio Co., Ltd., Sakado, Japan) containing 10% fetal bovine serum (Thermo Fisher Scientific) and 1% penicillin/streptomycin (Figure 3). Passage 2 cells were cryopreserved after resuspension in CELL BANKER^®^ 1plus (Takara Bio Inc., Kusatsu, Japan).

### Decellularized Scaffold Implantation Surgery

**FIGURE 4:**
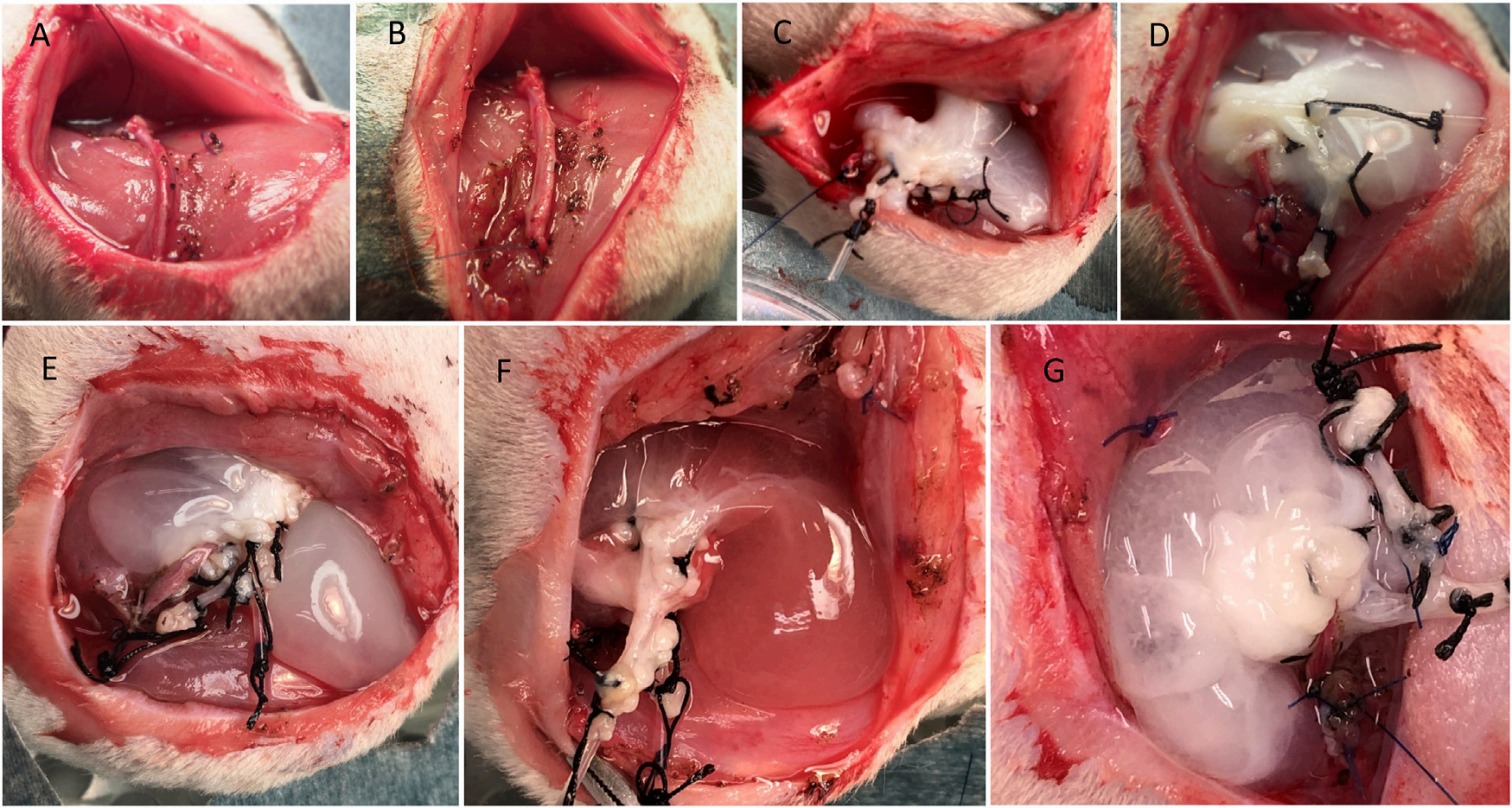
Steps of the DLM implantation. **(A)** AV bundle dissection, **(B)** ligation of the AV bundle, **(C)** implantation of the liver in the left thigh region of Lewis rat and passage of AV bundle, **(D)** the final configuration of the DLM and AV bundle, **(E, F, G)** injection of HBSS, fresh BM, or ADSCs into the scaffold through the portal vein, respectively.

Fourteen-to 16-week-old male Lewis rats were used as recipients (315–370 g). Induction of anesthesia was achieved using 3–4% isoflurane inhalation, and 1.5% isoflurane was used for maintenance during surgery. After hair clipping, the skin of the lower abdomen and left thigh region was prepared with 70% alcohol, and a sterile surgical drape was sutured around the location. A curved skin incision was performed, and the saphenous vessels were dissected using microsurgical techniques and electrocautery. Heparin sodium (100 U) was injected intravenously, followed by ligation of the arteriovenous (AV) bundle at the knee level (before bifurcation). The liver was placed inside the subcutaneous pouch, and the AV bundle was threaded through the scaffold’s IVC to enter from the supra-hepatic IVC and exit through the infra-hepatic IVC by passing the AV bundle ligation thread through the IVC tube. The IVC tube was then removed, and the AV bundle was fixed to the muscle using 5/0 nylon sutures. The scaffold was oriented, and a drop of Viti-bond glue (3M, St. Paul, MN, United States) was used to keep the scaffold in place (Figures 4A–D). The cannulas to the PV and HA were cut and kept inside the subcutaneous pocket for future use. Hemostasis was checked, and the wound was closed in 2 layers using 4/0 Nylon sutures. Saline (5 ml) containing 100 µL of 30% glucose was injected subcutaneously for rehydration. Rats were kept in a warm cage until complete recovery.

Before wound closure, 2.5 ml of HBSS (Thermo Fisher Scientific) was injected through the PV cannula using a butterfly 23-G needle containing no cells (HBSS group; *n* = 4), freshly obtained BM preparation (BM group; *n* = 3), or ADSCs (ADSC group; *n* = 3). Injection was manually performed at a rate of 1 ml/min (Figures 4E–G).

### Fresh BM Collection and Transplantation

BM was collected from the femur of female Lewis rats after decontamination with 1% Isodine solution (Mundipharma, Tokyo, Japan). Using sterile instruments, the femur was cut near its end, and BM was extruded onto a cell culture plate using HBSS. The process was repeated for the other femur (5 ml total). The collected crude BM was then gently aspirated using a 25-G butterfly cannula for mechanical dispersion to produce a single-cell suspension and collected into a sterile 5-ml Eppendorf tube (Figure 3). A 2.5-ml aliquot of the single-cell suspension was injected into the decellularized scaffold through the PV. Cell counting was then performed using a hemocytometer.

### ADSC Collection and Transplantation

The cryopreserved ADSCs were thawed at 37°C, resuspended in 10 ml of ADSC-1 medium, and centrifuged at 400 *g* for 5 min at 4°C. The supernatant was discarded, and the pellet was resuspended in medium. The cells were cultured in T-75 flasks. The medium was changed every two to 3 days. Before the time of transplantation, the ADSCs from passage 3 or 4 were collected after detachment with 0.05% trypsin/EDTA (Thermo Fisher Scientific) and washed. A total of 4×10^6^ cells were then suspended in 2.5 ml of HBSS, and the 2.5-ml cell suspension was injected into the decellularized scaffold through the PV. The ADSCs used in this study belong to the same lot (from the same isolation).

### Follow-up During the Pre-Vascularization Period

After surgery, the rats were followed up by body weight measurements until day 3 and then weekly for 4 weeks. Four weeks after surgery, streptozotocin (STZ; Sigma-Aldrich) was injected intravenously through the penile vein (65 mg/kg). The blood glucose was monitored using a portable glucometer (Freestyle; ABBOTT, Tokyo, Japan). Rats were considered diabetic if 2 consecutive measures of blood glucose were >400 mg/dl.

### Pancreatic Islet Isolation and Transplantation

Pancreatic islets were isolated from male Lewis donors as described previously (Saitoh et al., 2021). In brief, rats were anesthetized by isoflurane inhalation (Abbott Japan Co., Ltd., Tokyo, Japan). The bile duct was identified and clamped at the papilla of Vater. Cold HBSS (10 ml) containing 1 mg/ml collagenase (Sigma type V; Sigma Chemicals, St. Louis, MO, United States) was injected into the common bile duct leading to the pancreas. The pancreas was removed and incubated in a water bath at 37°C for 12 min before being digested, and the cell suspension was washed 3 times in cold HBSS and centrifuged for 1 min. Density-gradient centrifugation was performed for 10 min using a Histopaque-1119 (Sigma Diagnostics, St. Louis, MO, United States) and LymphoprepTM (Axis-Shiled, Oslo, Norway) to isolate pancreatic islets. The islets were cultured in Roswell Park Memorial Institute Medium 1640 (RPMI-1640) (Thermo Fisher Scientific) containing 5.5 mmol/L glucose, 10% fetal bovine serum (Thermo Fisher Scientific), and 1% penicillin/streptomycin (Thermo Fisher Scientific) at 37°C in 5% CO_2_ and humidified air (Figure 3).

One day after isolation, the islets were collected for transplantation. Anesthesia and surgical site preparation were performed as described earlier. The previous incision was re-opened, and the scaffold was exposed and carefully dissected from the overlying fibrofatty tissue. The cannula to the PV was exposed, and 300 µL of normal saline was injected using a 25-/23-G butterfly needle connected to a 1-ml syringe to test the patency. A total of 2,600 islet equivalents (IEQs; approximately 8 IEQs/g of rat weight) were then transferred to 100 µL of saline and aspirated using the butterfly needle. The islets were slowly injected into the scaffold through the PV in a total volume of 0.5–1 ml of saline and at a rate of 1 ml/min (manually). Occasional slight leakage of clear fluid out of the scaffold was considered acceptable.

The wound was then closed, and the rat was allowed to recover as described earlier. In one extra rat, both islets and hepatocytes (freshly isolated from male Lewis rats as described previously (Saitoh et al., 2021)) were injected, and the scaffold was retrieved immediately after injection to check the distribution of cells at day 0.

### Follow-up after Islet Transplantation

The rats were followed up for 30 days (±2 days) after transplantation for their general condition and correction of hyperglycemia. Rats were considered cured if 2 consecutive glucose measures were below 200 mg/dl. In cured animals and those with partial correction of hyperglycemia, an intravenous glucose tolerance test (IVGTT) was performed on day 28, and the scaffold was removed to confirm recurrence of hyperglycemia. The scaffold was fixed in 4% paraformaldehyde overnight and used for immunohistochemical analyses. 5-bromo-2′-deoxyuridine (BrdU; Abcam, Cambridge, United Kingdom) was injected intraperitoneally (i.p.) on days -4, -3, and -2 relative to the scaffold removal day (dose = 500 mg/kg). In one rat per group, micro-computed tomography (micro-CT) was performed at the study end-point to evaluate the AV bundle, as described later.

### IVGTT

The IVGTT was performed as described previously (Jimbo et al., 2014; Inagaki et al., 2021). In brief, after fasting for 14 h with free access to water, the BW and BGL were measured, and 1 g/kg glucose was injected intravenously. The blood glucose was measured at 5, 10, 20, 30, 60, 90, and 120 mins, and the blood glucose curve was generated. The area under the curve (AUC) was then used for comparisons.

### Ex Vivo Micro-CT

Μicro-CT was performed in one rat per group as described previously (Eweida et al., 2018a), with a few modifications. The abdominal aorta was cannulated with a 22-G venflon cannula, and 100 ml of normal saline containing 100 U of heparin sodium per mL was perfused using gravity to wash out the blood. A total of 20 ml of Microfil^®^ (MV-122; Flow Tech Inc., Carver, MA, United States) with 0.6 ml of curing agent was then injected using a 30-ml syringe through the cannula. After 90 min, the implanted scaffold was dissected and carefully removed. The scaffold was then imaged using a LaTheta™ LCT-200 (Hitachi Aloka Medical Ltd., Tokyo, Japan). The VGSTUDIO MAX™ software program (version 3.0) (Volume Graphics, Heidelberg, Germany) was used for the 3D reconstruction and video acquisition.

### Immunohistochemistry

Removed scaffolds were fixed in 4% paraformaldehyde, dehydrated, and embedded in paraffin blocks. In brief, 4-μm sections were incubated with rabbit anti-von-Willebrand factor (anti-vWF;Sigma Aldrich, ab7356), rabbit anti-insulin (Abcam, ab181547), and rat anti-BrdU antibody-HRP (Abcam, ab220507). Rabbit Envision and goat anti-rabbit-HRP (4003; DAKO, Glostrup, Denmark) were used as secondary antibodies for anti-vWF and anti-insulin staining, respectively. Either methylene green or Hematoxylin was used for nuclear staining. Hematoxylin-Eosin (HE) staining was also performed. Six to 8 sections with equal intervals (250 µm) were evaluated per sample and scored by a pathologist in a blinded manner. The positive cells for vWF and BrdU were counted, and the total islet area was measured in each section. Then, we calculated the number of positive cells for vWF and BrdU per µm^2^ of islet area (number/µm^2^) in each section. The resulting scores were used for statistical comparison.

### Statistical Analyses

Data for histological scoring are expressed as the mean ± standard error of mean (SEM). All statistical analyses were performed using the JMP pro 15 software program (SAS institute Inc., Carry, NC, United States). For histological scoring, a one-way analysis of variance (ANOVA) was used to compare the means, and the Tukey-Kramer test was used for the post hoc analysis. A student t-test was used to compare the means of AUC from the IVGTT results. *p* values of <0.05 were considered to indicate statistical significance.

## Results

### Gross Morphology

At the end of the decellularization protocol, the DLM demonstrated a whitish, transparent appearance (Figure 2B). With adequate dissection of the subcutaneous plane up to the inguinal ligaments, placement of the whole-liver decellularized scaffold was possible. At the time of islet transplantation, no significant adhesion or fibrous capsule was observed, and the scaffold was easily visualized. A considerable yet expected decrease in size and change in shape was noted at this point. Injection of pancreatic islets into the scaffold was feasible with minimal leakage.

**FIGURE 5:**
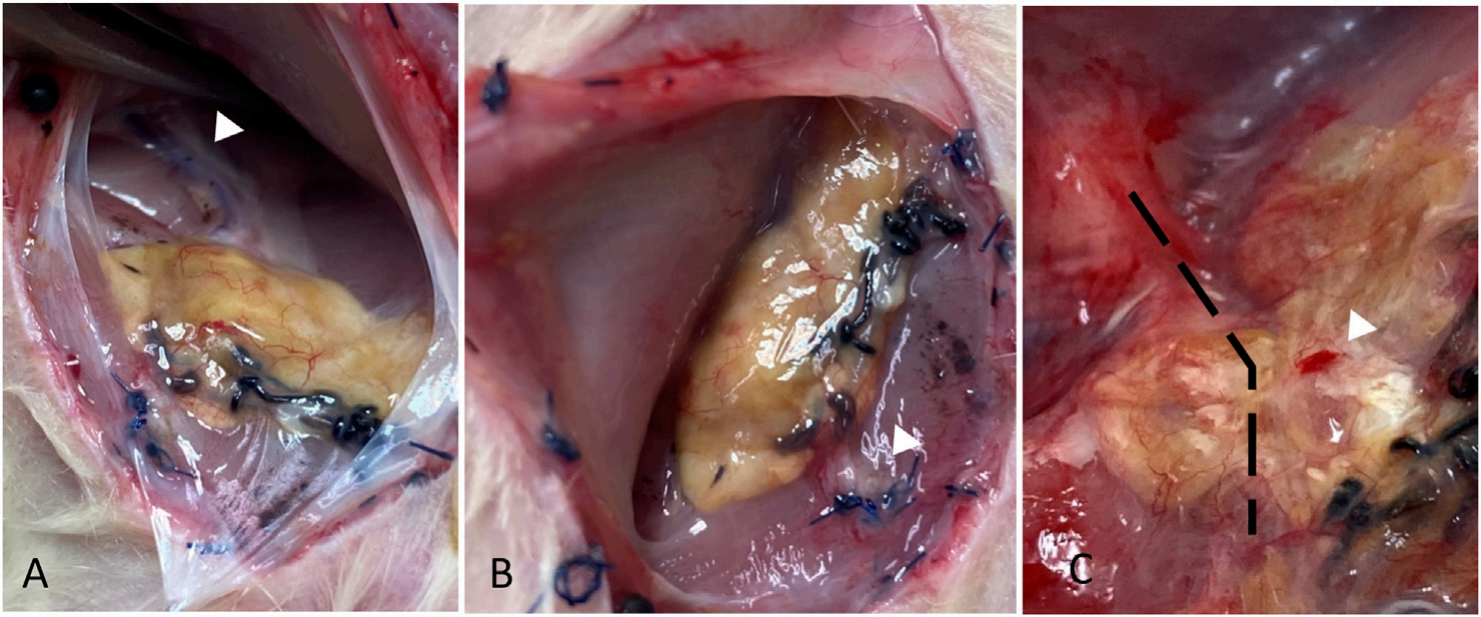
Gross morphology of the liver at the study end-point showing the AV bundle proximal and distal to the scaffold (white arrowheads in **A** and **B**, respectively), surface vascularization, and bleeding on touch (white arrowhead in **C**). The location of AV bundle in (**C**) is marked by a dashed line.

At the time of scaffold retrieval, the size of the scaffold was comparable to the size at islet transplantation. Vascularization of the scaffold surface was visible in most specimens. The AV bundle above and below the scaffold was clearly seen, and pulsation was frequently observed through the scaffold (Figure 5 and Supplementary Video 1). Cut sections through the scaffold showed an intact AV bundle, and the intra-graft vasculature was observed in silicon-injected samples.

### Blood Glucose Level

**FIGURE 6:**
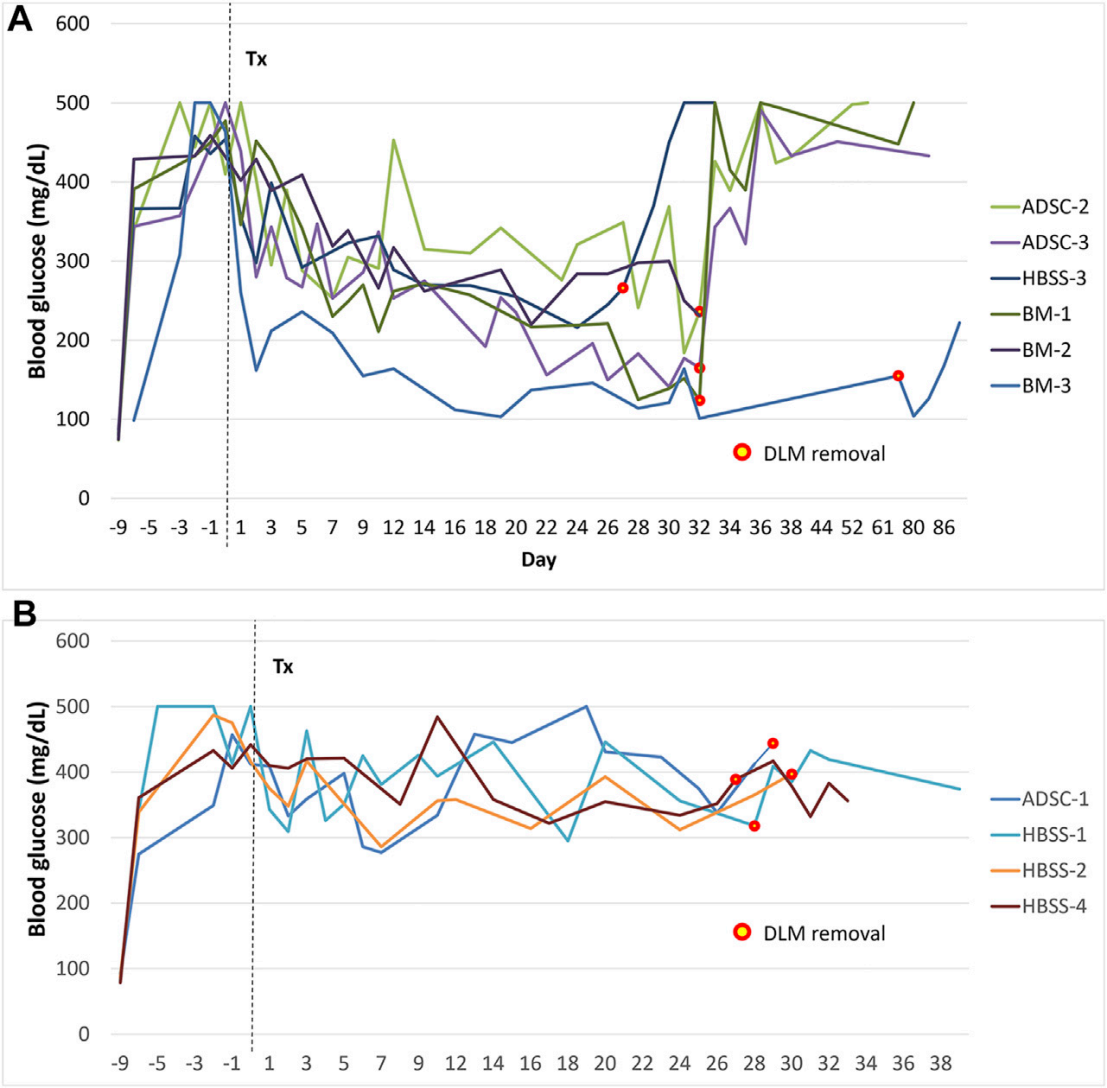
Blood glucose levels during follow-up after transplantation. **(A)** animals with improvement of blood glucose levels, **(B)** animals that did not show improvement of blood glucose levels. The mark signifies the time of graft removal. *The graft was removed after 81 days in the first rat of the study to check for long-term adverse effects (BM group, this graft was not included in histological or vascularization assessments).

Two rats in the BM group and 1 in the ADSC group showed normalization of blood glucose, (blood glucose <200 mg/dl on 2 consecutive measurements). One rat in each group showed partial correction of glucose level. No rats were cured in the HBSS group. After graft removal, hyperglycemia recurred, confirming the function of the graft. In one rat in the BM group, hyperglycemia did not completely recur after scaffold removal. Figure 6 shows the blood glucose levels of the rats during the study period.

**FIGURE 7:**
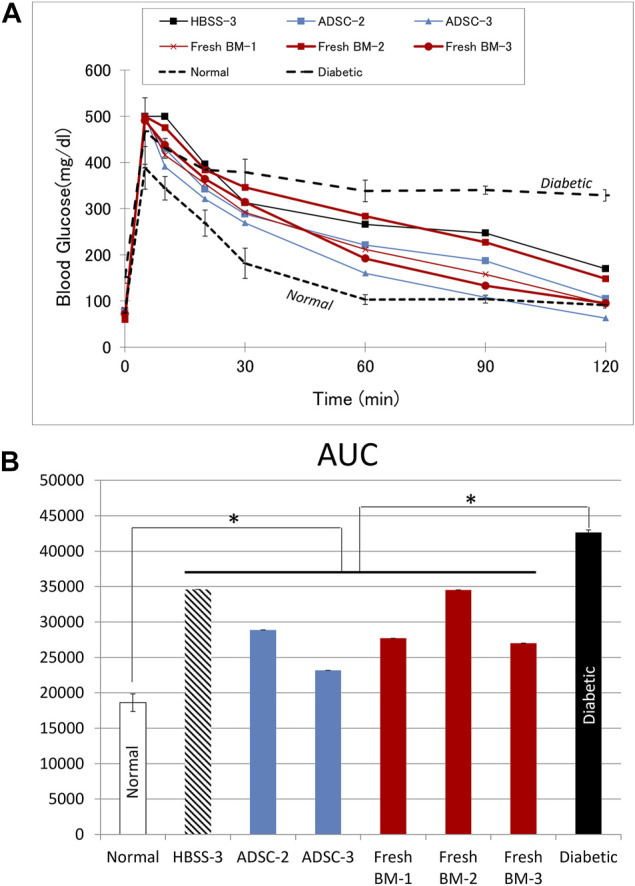
Intravascular glucose tolerance test (IVGTT). **(A)** The blood glucose curve in animals with improved blood glucose levels. **(B)** The corresponding area under the curve (AUC). Average data from diabetic (*n* = 3) or normal (*n* = 6) Lewis rats are provided for comparison. In comparison to diabetic rats, AUC was significantly lower (*p* = 0.002) in the treatment group with functioning graft, while it was significantly higher (*p* < 0.001) in comparison to values from normal rats.

The IVGTT was performed in rats with improved blood glucose levels (*n* = 6), and the results are shown in Figure 7. The AUC in rats with functioning grafts were significantly lower than values in diabetic rats (*n* = 3), *p* = 0.002, while significantly higher compared to normal Lewis rats (*n* = 6), *p* < 0.001.

### Micro-CT

**FIGURE 8:**
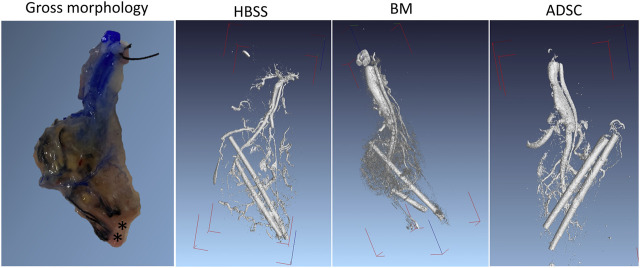
*Ex vivo* micro-CT imaging. *Ex vivo* micro-CT was performed in one rat per group after injecting Microfil through the abdominal aorta. The DICOM files were reconstructed using a visual studio software program. The PV and HA cannulas were left in place for orientation (radioopaque). The gross morphology of the graft after Microfil injection is provided for anatomical correlation (the asterixis mark the cannulas’ location).

In all three groups, there was evidence of vascular sprouting from the AV bundle inside the scaffold. Figure 8 shows representative micro-CT 3D reconstruction images. The micro-CT examination also confirmed the patency of the AV bundle in samples from the three groups (HBSS, BM, and ADSC). A representative video through the axial scans can be found in the supplementary data (Supplementary Video 2).

### Immunohistochemistry

**FIGURE 9:**
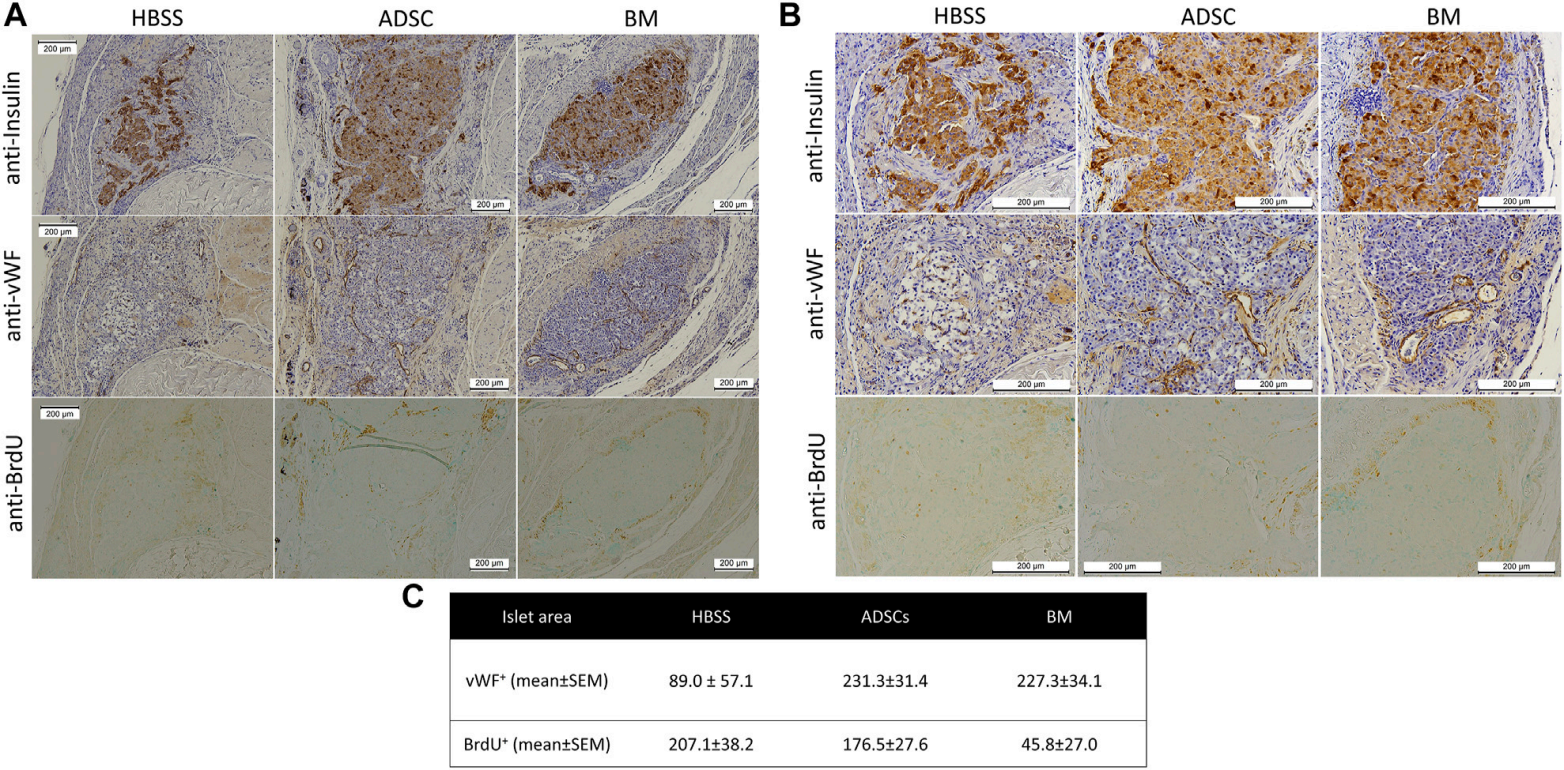
Representative images of the immunohistochemical staining of the scaffold showing anti-insulin **(upper panel),** anti-Von-Willebrand factor **(middle panel),** and anti-BrdU **(lower panel)** staining of the islet region [**(A)** low magnification (100x) and **(B)** high magnification (200x)]. **(C)** There were more vWF + cells in the islet region in the ADSC and BM groups than in the HBSS group, while the numbers of BrDU + cells were significantly lower in the BM group than in the other two groups.

Histological examination showed insulin-positive cells in all three study groups (Figure 9). However, intact islets were more frequently encountered in the BM and ADSC groups than in the acellular scaffolds. The number of vWF-positive cells was higher within the islet region in the ADSC and BM groups than in the HBSS group (*p* = 0.08 and 0.10, respectively). BrdU + cells were significantly less frequent in the BM group than in both the ADSC and acellular groups (*p* = 0.003 for both), with no significant difference noted between the HBSS and ADSC groups.

**FIGURE 10:**
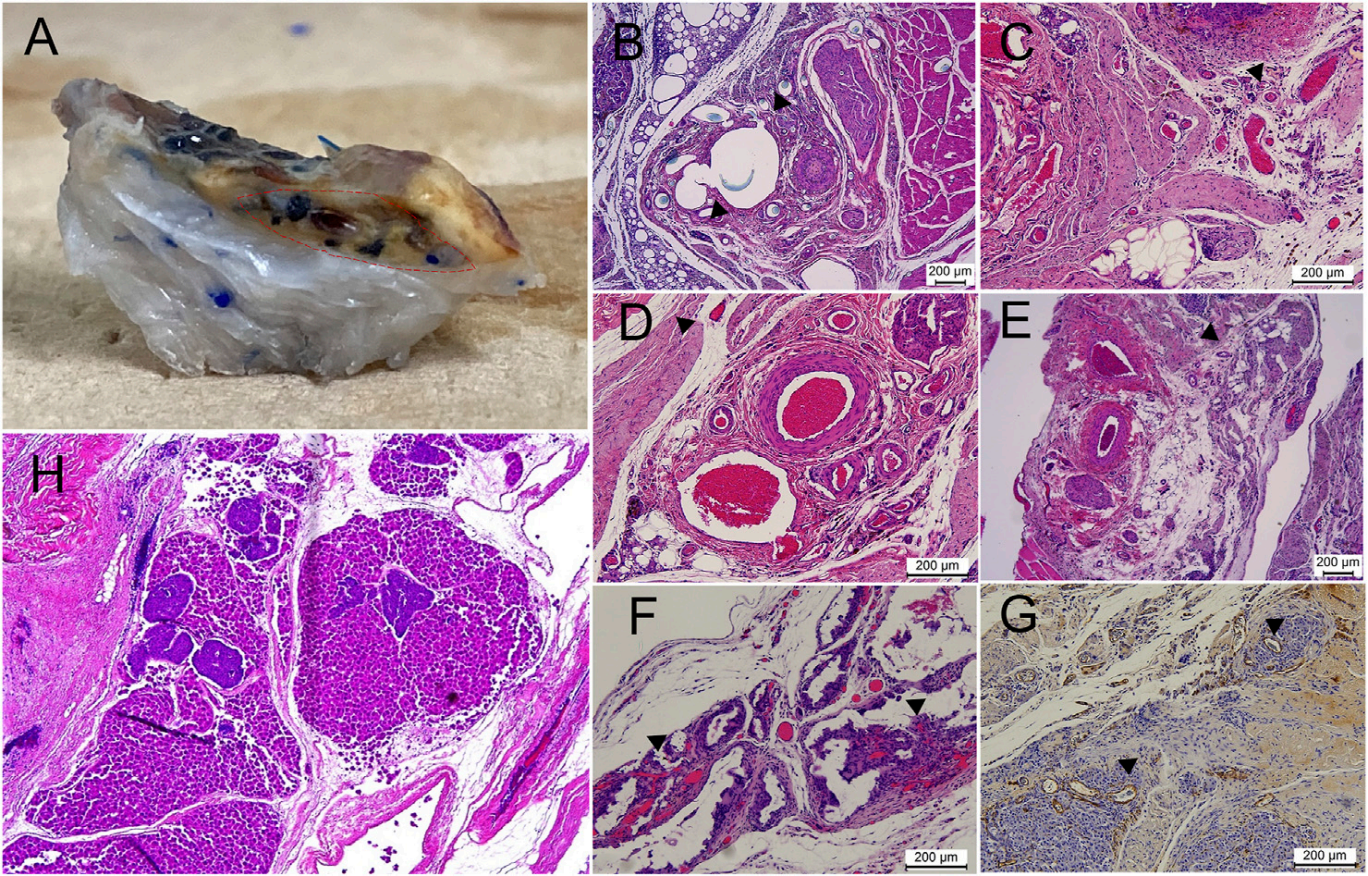
Vascularity of the implanted scaffold at the end of the study. **(A)** Large vessels inside the scaffold, shown by injection of colored dye (region encircled by dashed red line). B-G) Different patterns of vascularization seen on a histological examination, with the following findings marked with black arrowheads: **(B)** direct sprouting from the main vein of the AV bundle, **(C)** vessels observed inside the scaffold in close association with the AV bundle, **(D,E)** sprouting of vessels along the native scaffold inferior vena cava tributaries, **(F)** remarkable intra-scaffold vascularization in association with scaffold resorption regions, **(G)** anti-Von-Willebrand staining of neovascularization inside the islet graft. **(H)** Day 0 specimen after combined injection of both pancreatic islets and hepatocytes; islets frequently failed to reach the intra-scaffold location compared with smaller hepatocytes.

The AV bundle was intact and patent in all analyzed samples with evident sprouting into the scaffold (Figure 10). No evidence of fibrosis or capsule formation was noted. A honeycomb appearance of the empty DLM was noted in some regions, while evidence of resorption could also be seen at other locations.

## Discussion

Decellularized ECM scaffolds were shown to support the islet function *in vitro* (Napierala et al., 2017; Guruswamy Damodaran and Vermette, 2018; Citro et al., 2019). In addition, transplanting islets with a decellularized lung scaffold was functionally superior to islets-only when transplanted in the subcutaneous tissue (Citro et al., 2019). In another report (Yu et al., 2018), a recellularized pancreas scaffold improved the blood glucose levels when anastomosed to diabetic rat vessels, although the graft survival was short. A decellularized pancreatic scaffold accelerated the insulin gene expression in induced pluripotent stem cell-derived β-cell-like cells (Wan et al., 2017). In the present proof-of-concept study, we showed that decellularized whole liver can allow for islet engraftment and the graft survival in a rat model. The choice of the subcutaneous location, known for its low vascularity and oxygen tension, and islets, with their high oxygen demand, allowed us to examine the potential utility of this approach for cell transplantation.

Several studies have demonstrated the utility of the axial vascularization concept in tissue-engineered constructs and its ability to promote vascularized tissue formation (Eweida et al., 2018a; Eweida et al., 2018b; Wong et al., 2019). However, few studies have examined the suitability of axially vascularized chambers for islet transplantation in the form of an AV shunt (Maki et al., 1996; Brown et al., 2006) or bundle (Knight et al., 2006). Results have shown an improved glycemic control compared to diabetic controls (Maki et al., 1996; Knight et al., 2006) as well as evidence of intact islets on histology (Brown et al., 2006). Tanaka et al. (Tanaka et al., 2003) compared the AV bundle to AV shunt and found a significantly higher rate of new tissue formation in cases with an AV shunt. However, excessive vascular sprouting was noted in both the AV shunt and bundle (ligation type) groups. Because of its simplicity and suitability for the scaffold size and orientation, a ligation-type AV bundle was chosen for the present study.

Both micro-CT and the histological assessment showed intact AV bundles more than 65 days after the initial scaffold implantation. Furthermore, sprouting from the AV bundle was clearly seen on histology and micro-CT. Branching along the native IVC tributaries and, to a lesser extent, direct penetration through the IVC-ECM could be seen during the histological examination (Figure 10). The AV bundle was passed through the IVC for two reasons: because the grossly multi-lobular nature of the rat liver makes it difficult to pass the bundle through all the lobes, and to avoid damaging the integrity of the scaffold to allow for seeding through the PV later. The histological assessment showed that more vWF-positive vessels were found in the BM and ADSC groups than HBSS group, while no superiority was noted with regard to BrdU staining, suggesting that the improvement in the function was likely due to better islet vascularization.

The subcutaneous location offers many advantages for islet transplantation including easy accessibility, low invasiveness, and monitoring feasibility. However, due to its poor vascularization, a pre-vascularization approach is usually necessary in order to achieve an islet function comparable to that with intra-portal transplantation (Uematsu et al., 2018a; Uematsu et al., 2018b). In this approach, a device is placed in the subcutaneous tissue to induce a vascular capsule, and then islets are transplanted inside this capsule after a certain period. This device is either made of material that induces neovascularization or loaded with angiogenic growth factors (Uematsu et al., 2018a; Uematsu et al., 2018b). In the present study, we examined the effect of ECM alone compared with seeded ECM with fresh BM or ADSCs to induce vascularization of the scaffold.

Several studies have demonstrated the angiogeneic potential of both ADSCs and BM-derived mesenchymal stem cells (BM-MSCs) (reviewed in (Mazini et al., 2019; Liu and Holmes, 2021)) and their ability to improve the islet function (Jung et al., 2011; Chen et al., 2013; Lee et al., 2020; Yamashita et al., 2021). One study demonstrated a better vascularization after ADSC implantation than with BM-MSCs in a myocardial infarction (MI) model (Paul et al., 2013). Extracellular vesicles (ECVs) from ADSCs also had a superior effect compared to those from BM-MSCs in an MI model (Xu et al., 2020). In contrast, in two separate studies involving a brain-ischemia model, ECVs from BM-MSCs resulted in a 4-fold increase in the endothelial cell number (Doeppner et al., 2015) compared to a 1.5-fold increase in the other study utilizing ADSCs (Chen et al., 2016). While a direct comparison between BM-MSCs and ADSCs was not the target of the present study, with these cells instead compared to an acellular scaffold, our findings suggest that ADSCs did not offer any particular advantage over fresh BM (MSCs ≈0.004–0.009% of nucleated cells in murine BM (Meirelles and Nardi, 2003)) with regard to the islet function or number of newly formed vessels. Co-transplantation of either cell type alone without a pre-vascularization device was not reported to be successful in allowing the islet function in the subcutaneous tissue, although transplantation with ADSC-sheets showed promising results (Lee et al., 2020; Yamashita et al., 2021). *In vitro,* ADSC-sheet + islets co-culture media showed significantly higher concentration of IL-8 compared to control (Yamashita et al., 2021). A previous report showed that transplanting islets with decellularized small intestine submucosa (SIS) or with SIS + BM-MSCs resulted in significantly lower blood glucose levels, although the latter showed significantly higher blood insulin levels (Wang et al., 2017). The study showed that co-culturing with BM-MSCs significantly increased vascular endothelial growth factor A (Vegfa) expression and CD31 mean fluorescence intensity in islets compared to islets-only and islets + SIS groups *in vitro*. The co-culture media also had significantly higher concentrations of ciliary neurotrophic factor (CNTF), epidermal growth factor (EGF), hepatocyte growth factor (HGF) (Wang et al., 2017).

In addition to improving islet revascularization, BM-MSCs (Chen et al., 2013; Wang et al., 2017; He et al., 2018) and ADSCs (Lee et al., 2020; Yamashita et al., 2021) were shown to improve islet viability and stimulated insulin secretion *in vitro*. BM-MSCs also significantly upregulated insulin 1 (Ins1) and pancreatic and duodenal homeobox 1 (Pdx1) expression in islets (Wang et al., 2017). BM-MSCs co-culture protected the islets against endoplasmic reticulum stress-induced apoptosis in another study (He et al., 2018). The concentration of tumor necrosis factor (TNF), in BM-MSC + islets co-culture media (Wang et al., 2017), and IL-16, in ADSCs + islets co-culture media (Yamashita et al., 2021), were significantly lower in comparison to islets-only medium.

The merits of the present approach include its functionality in the subcutaneous location, feasibility of repeated transplantation and drug delivery, accessibility for removal or re-implantation through the AV bundle without animal sacrifice, allowance for ECM material modifications after decellularization, and suitability for studying cell-ECM interactions. However, the main demerit of this approach is the active ECM resorption, although this can be considered a part of the remodeling process. Therefore, the balance between the pre-vascularization period and the expected ECM resorption should be tuned according to the size of the scaffold. In addition, a day 0 assessment showed that not all islets could reach an intraparenchymal location, and some got stuck at the terminal portal venules. This finding is supported by a previous report (Fu et al., 2017) and justifies the transplantation of many islets in this study. Accordingly, this model might be more suitable for single-cell preparations (e.g., hepatocytes or disintegrated beta cells) or small organoids (small islets or hepatocyte spheroids). If the challenges associated with upscaling could be overcome, this model can be a promising alternative site for clinical cell transplantation and a novel approach for organ bioengineering.

In conclusion, despite the limited sample size, the study demonstrated the potential utility of the pre-vascularized whole-organ scaffold as a novel approach for islet transplantation. Both BM- and ADSCs-seeded scaffolds were superior to the acellular scaffold with regard to the vascularization and graft function.

## Data Availability

All data generated or analyzed in the present study were included in this published article and its supporting files.

## Supplementary Material

Supplementary Video 1 Arterial pulsations.

Supplementary Video 2 Micro-CT.

## Abbreviations

ADSCs, adipose-derived stem cells; AUC, area under the curve; AV, arteriovenous; BM, bone marrow; BrdU, 5-bromo-2′-deoxyuridine; CNTF, ciliary neurotrophic factor; CT, computed tomography; DLM, decellularized liver; DW, distilled water; ECM, extracellular matrix; ECV, extracellular vesicles; EGF, epidermal growth factor; HA hepatic artery; HBSS, Hank’s balanced salt solution; HGF, hepatocyte growth factor; IEQ, islet equivalent; Ins1, insulin 1; IVC, inferior vena cava; IVGTT, intravenous glucose tolerance testi.p., intraperitoneal; MI, myocardial infarction; MSCs, mesenchymal stem cells; PBS, phosphate buffered saline; Pdx1, pancreatic and duodenal homeobox; 1PV, portal vein; RPMI, Roswell Park Memorial Institute; SDS, sodium dodecyl sulphate; SIS, small intestinal submucosa; STZ, streptozotocin; TNF, tumor necrosis factor; Vegfa, vascular endothelial growth factor; WF, von-Willebrand factor.
